# Hypercycle

**DOI:** 10.1371/journal.pcbi.1004853

**Published:** 2016-04-07

**Authors:** Natalia Szostak, Szymon Wasik, Jacek Blazewicz

**Affiliations:** 1 Institute of Computing Science, Poznan University of Technology, Poznan, Poland; 2 European Center for Bioinformatics and Genomics, Poznan University of Technology, Poznan, Poland; 3 Institute of Bioorganic Chemistry, Polish Academy of Sciences, Poznan, Poland; University of Toronto, CANADA

This is a “Topic Page” article for *PLOS Computational Biology*.

## Introduction

A hypercycle is an abstract model of organization of self-replicating molecules connected in a cyclic, autocatalytic manner. It was introduced in an ordinary differential equation (ODE) form by the Nobel Prize winner Manfred Eigen in 1971 [1] and subsequently further extended in collaboration with Peter Schuster [2,3]. It was proposed as a solution to the error threshold problem encountered during modelling of replicative molecules that hypothetically existed on the primordial Earth (see: abiogenesis). The hypercycle is a special case of the replicator equation [4]. The most important properties of hypercycles are autocatalytic growth competition between cycles, once-for-ever selective behaviour, utilization of small selective advantage, rapid evolvability, increased information capacity, and selection against parasitic branches.

**Fig 1:**
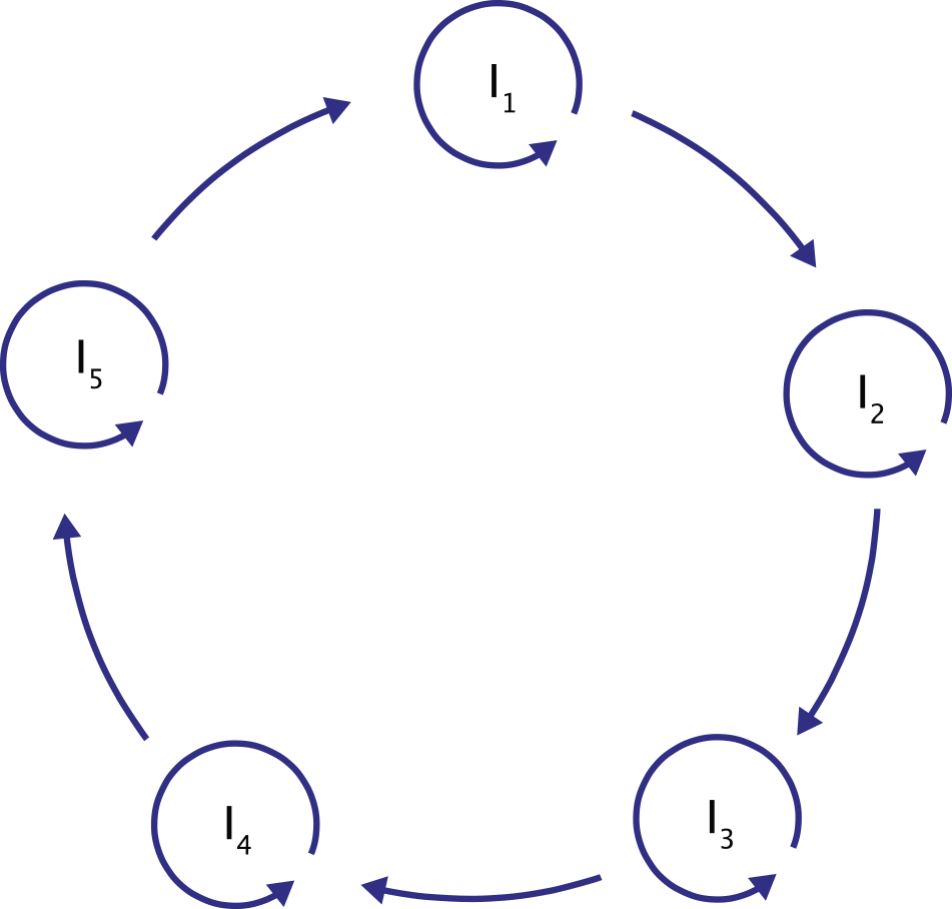
A hypercycle.

The hypercycle is a cycle of connected, self-replicating macromolecules. In the hypercycle, all molecules are linked such that each of them catalyses the creation of its successor, with the last molecule catalysing the first one. In such a manner, the cycle reinforces itself (Fig 1). Furthermore, each molecule is additionally a subject for self-replication. The resultant system is a new level of self-organization that incorporates both cooperation and selfishness. The coexistence of many genetically non-identical molecules makes it possible to maintain a high genetic diversity of the population. This can be a solution to the error threshold problem, which states that, in a system without ideal replication, an excess of mutation events would destroy the ability to carry information and prevent the creation of larger and fitter macromolecules. Moreover, it has been shown that hypercycles could originate naturally and that incorporating new molecules can extend them. Hypercycles are also subject to evolution and, as such, can undergo a selection process. As a result, not only does the system gain information, but its information content can be improved. From an evolutionary point of view, the hypercycle is an intermediate state of self-organization, but not the final solution.

Over the years, the hypercycle theory has experienced many reformulations and methodological approaches. Among them, the most notable are applications of partial differential equations [5], cellular automata [6–9], and stochastic formulations of Eigen’s problem [10,11]. Despite many advantages that the concept of hypercycles presents, there were also some problems regarding the traditional model formulation using ODEs: a vulnerability to parasites and a limited size of stable hypercycles [12,13]. In 2012, the first experimental proof for the emergence of a cooperative network among fragments of self-assembling ribozymes was published, demonstrating their advantages over self-replicating cycles [14]. However, even though this experiment proves the existence of cooperation among the recombinase ribozyme subnetworks, this cooperative network does not form a hypercycle per se, so we still lack the experimental demonstration of hypercycles [15].

## Model Formulation

### Error threshold problem

**Fig 2:**
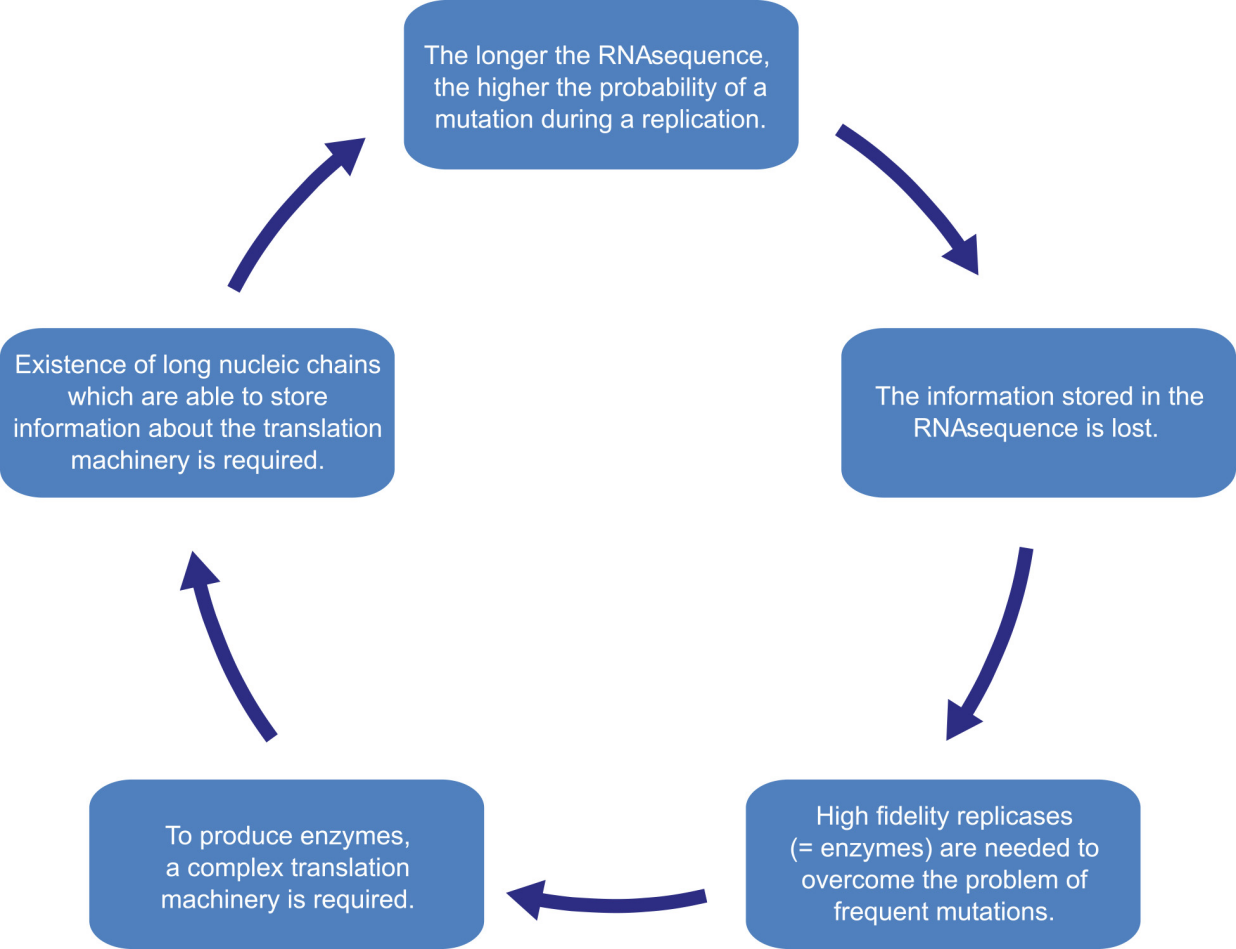
A presentation of the error threshold problem.

When a model of replicating molecules was created [1,2], it was found that, for effective storage of information, macromolecules on prebiotic Earth could not exceed a certain threshold length. This problem is known as the error threshold problem. It arises because replication is an imperfect process, and during each replication event, there is a risk of incorporating errors into a new sequence, leading to the creation of a quasispecies. In a system that is deprived of high-fidelity replicases and error-correction mechanisms, mutations occur with a high probability. As a consequence, the information stored in a sequence can be lost due to the rapid accumulation of errors, a so-called error catastrophe. Moreover, it was shown that the genome size of any organism is roughly equal to the inverse of mutation rate per site per replication [16–18]. Therefore, a high mutation rate imposes a serious limitation on the length of the genome. To overcome this problem, a more specialized replication machinery that is able to copy genetic information with higher fidelity is needed. Manfred Eigen suggested that proteins are necessary to accomplish this task. However, to encode a system as complex as a protein, longer nucleotide sequences are needed, which increases the probability of a mutation even more and requires even more complex replication machinery. This vicious circle is known as Eigen’s Paradox (Fig 2) [3].

According to current estimations, the maximum length of a replicated chain that can be correctly reproduced and maintained in enzyme-free systems is about 100 bases [19], which is assumed to be insufficient to encode replication machinery. This observation was the motivation for the formulation of the hypercycle theory.

### Hypercycle models

It was suggested that the problem with building and maintaining larger, more complex, and more accurately replicated molecules can be circumvented if several information carriers, each of them storing a small piece of information, are connected such that they only control their own concentration [1,2]. Studies of the mathematical model describing replicating molecules revealed that to observe a cooperative behaviour among self-replicating molecules, they have to be connected by a positive feedback loop of catalytic actions [20–22]. This kind of closed network consisting of self-replicating entities connected by a catalytic positive-feedback loop was named an elementary hypercycle (Fig 1). Such a concept, apart from an increased information capacity, has another advantage. Linking self-replication with mutual catalysis can produce nonlinear growth of the system. This, first, makes the system resistant to so-called parasitic branches. Parasitic branches are species coupled to a cycle that do not provide any advantage to the reproduction of a cycle, which, in turn, makes them useless and decreases the selective value of the system. Secondly, it reinforces the self-organization of molecules into the hypercycle, allowing the system to evolve without losing information, which solves the error threshold problem.

**Fig 3:**
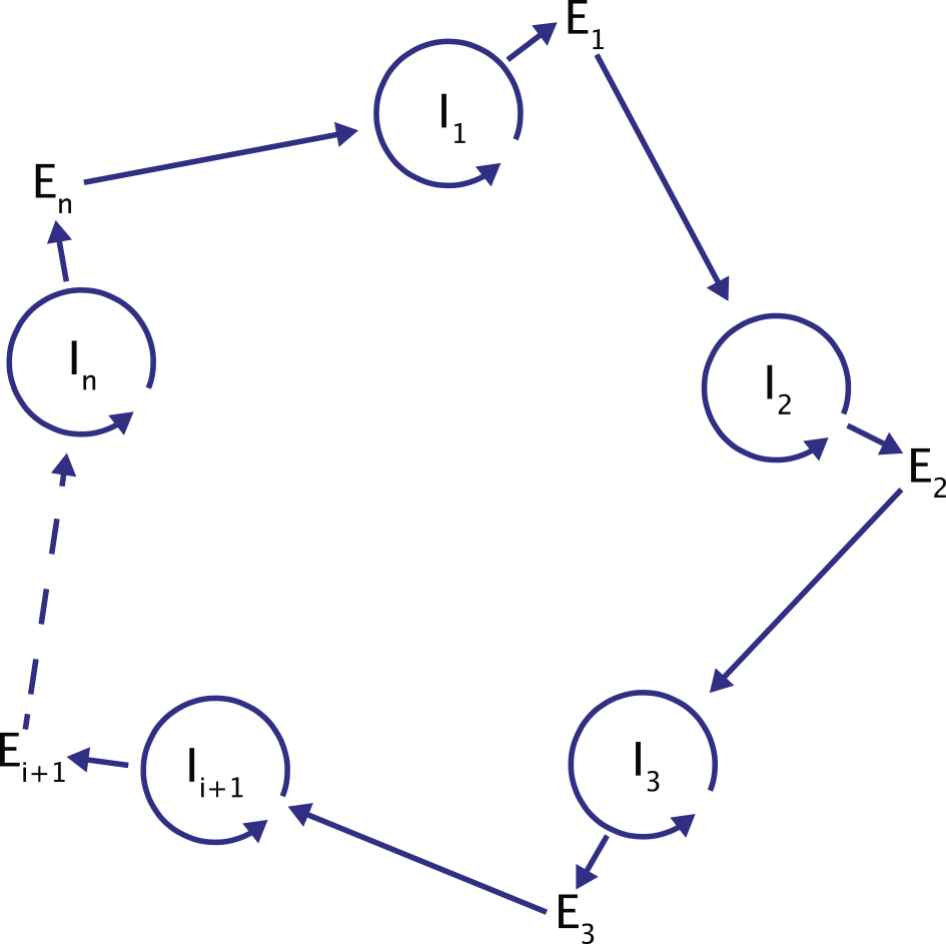
A hypercycle with translation.

Analysis of potential molecules that could form the first hypercycles in nature prompted the idea of coupling an information carrier function with enzymatic properties. At the time of the hypercycle theory formulation, enzymatic properties were attributed only to proteins, while nucleic acids were recognized only as carriers of information. This led to the formulation of a more complex model of a hypercycle with translation) (Fig 3). The proposed model consists of a number of nucleotide sequences I (I stands for intermediate) and the same number of polypeptide chains E (E stands for enzyme). Sequences I have a limited chain length and carry the information necessary to build catalytic chains E. The sequence I_*i*_ provides the matrix to reproduce itself and a matrix to build the protein E_*i*_. The protein E_*i*_ gives the catalytic support to build the next sequence in the cycle, I_i+1_. The self-replicating sequences I form a cycle consisting of positive and negative strands)that periodically reproduce themselves. Therefore, many cycles of the +/− nucleotide collectives are linked together by the second-order cycle of enzymatic properties of E, forming a catalytic hypercycle. Without the secondary loop provided by catalysis, I chains would compete and select against each other instead of cooperating. The reproduction is possible thanks to translation and polymerization functions encoded in I chains. In his principal work, Manfred Eigen stated that the E coded by the I chain can be a specific polymerase or an enhancer) (or a silencer)) of a more general polymerase acting in favour of formation of the successor of nucleotide chain I [1]. Later, he indicated that a general polymerase leads to the death of the system [2]. Moreover, the whole cycle must be closed, so that E_*n*_ must catalyse I_1_ formation for some integer *n* > 1.

### Alternative concepts

During their research, Eigen and Schuster [2] also considered types of protein and nucleotide coupling other than hypercycles. One such alternative was a model with one replicase that performed polymerase functionality and that was a translational product of one of the RNA matrices existing among the quasispecies. This RNA-dependent RNA polymerase catalysed the replication of sequences that had specific motifs recognized by this replicase. The other RNA matrices, or just one of their strands, provided translational products that had specific anticodons and were responsible for unique assignment and transportation of amino acids.

Another concept devised by Eigen and Schuster was a model in which each RNA template’s replication was catalysed by its own translational product; at the same time, this RNA template performed a transport function for one amino acid type. Existence of more than one such RNA template could make translation possible.

Nevertheless, in both alternative concepts, the system will not survive due to the internal competition among its constituents. Even if none of the constituents of such a system is selectively favoured, which potentially allows coexistence of all of the coupled molecules, they are not able to coevolve and optimize their properties. In consequence, the system loses its internal stability and cannot live on. The reason for inability to survive is the lack of mutual control of constituent abundances).

## Mathematical Model

### Elementary hypercycle

The dynamics of the elementary hypercycle can be modelled using the following differential equation [3]:

$${\dot{x}}_{i}=x_{i}\;\left(k_{i}+\sum_{j}k_{i,j}\;x_{j}-\frac{1}{x}\phi \right)$$

where

$$x=\sum_{i}x_{i}\;,$$

$$k_{i}=f_{i}-d_{i}.$$

In the equation above, *x*_*i*_ is the concentration of template I_*i*_; *x* is the total concentration of all templates; *k*_*i*_ is the excess production rate of template I_*i*_, which is a difference between formation *f*_*i*_ by self-replication of the template and its degradation *d*_*i*_, usually by hydrolysis; *k*_i,j_ is the production rate of template I_*i*_ catalysed by I_*j*_*;* and *ϕ* is a dilution flux), which guarantees that the total concentration is constant. Production and degradation rates are expressed in numbers of molecules per time unit at unit concentration (*x*_*i*_ = 1). Assuming that at high concentration *x* the term *k*_*i*_ can be neglected, and, moreover, in the hypercycle, a template can be replicated only by itself and the previous member of the cycle, the equation can be simplified to:

$${\dot{x}}_{i}=x_{i}\;\left(k_{i,i-1}x_{i-1}-\frac{1}{x}\phi \right)$$

Where, according to the cyclic properties, it can be assumed that:

$$k_{i,0}=k_{i,n},$$

$$x_{0}=x_{n}.$$

### Hypercycle with translation

A hypercycle with translation consists of polynucleotides I_*i*_ (with concentration *x*_*i*_) and polypeptides E_*i*_ (with concentration *y*_*i*_) [2]. It is assumed that the kinetics of nucleotide synthesis follows a Michaelis–Menten-type reaction scheme in which the concentration of complexes cannot be neglected. During replication, molecules form complexes I_*i*_E_i-1_ (occurring with concentration *z*_*i*_). Thus, the total concentration of molecules (*x*_*i*_^0^ and *y*_*i*_^0^) will be the sum of free molecules and molecules involved in a complex:

$$x_{i}^{0}=x_{i}+z_{i},$$

$$y_{i}^{0}=y_{i}+z_{i+1}.$$

The dynamics of the hypercycle with translation can be described using a system of differential equations modelling the total number of molecules:

$${\dot{x}}_{i}^{0}=f_{i}z_{i}-\frac{x_{i}^{0}}{c_{\text{I}}}\phi_{x},$$

$${\dot{y}}_{i}^{0}=k_{i}x_{i}-\frac{y_{i}^{0}}{c_{\text{E}}}\phi_{y}$$

where

$$c_{\text{I}}=\sum_{i}x_{i}^{0},$$

$$c_{\text{E}}=\sum_{i}y_{i}^{0}.$$

In the above equations, *c*_E_ and *c*_I_ are total concentrations of all polypeptides and all polynucleotides, *ϕ*_*x*_ and *ϕ*_*y*_ are dilution fluxes, *k*_*i*_ is the production rate of polypeptide E_*i*_ translated from the polynucleotide I_*i*_, and *f*_*i*_ is the production rate of polynucleotide I_*i*_ synthesised by the complex I_*i*_E_i-1_ (through replication and polymerization).

Coupling nucleic acids with proteins in such a model of hypercycle with translation demanded the proper model for the origin of translation code as a necessary condition for the origin of hypercycle organization. At the time of hypercycle theory formulation, two models for the origin of translation code were proposed by Crick and his collaborators [23]. These were models stating that the first codons were constructed according to either an RRY or an RNY scheme, in which R stands for the purine base, Y for pyrimidine, and N for any base, with the latter assumed to be more reliable. Nowadays, it is assumed that the hypercycle model could be realized by utilization of ribozymes without the need for a hypercycle with translation, and there are many more theories about the origin of the genetic code.

## Evolution of Hypercycles

### Formation of the first hypercycles

Eigen made several assumptions about conditions that led to the formation of the first hypercycles [2]. Some of them were the consequence of the lack of knowledge about ribozymes, which were discovered a few years after the introduction of the hypercycle concept [24,25] and negated Eigen’s assumptions in the strict sense. The primary of them was that the formation of hypercycles had required the availability of both types of chains: nucleic acids forming a quasispecies population and proteins with enzymatic functions. Nowadays, taking into account the knowledge about ribozymes, it may be assumed that a hypercycle’s members were selected from the quasispecies population and the enzymatic function was performed by RNA. According to the hypercycle theory, the first primitive polymerase emerged precisely from this population. As a consequence, the catalysed replication could exceed the uncatalysed reactions, and the system could grow faster. However, this rapid growth was a threat to the emerging system, as the whole system could lose control over the relative amount of the RNAs with enzymatic function. The system required more reliable control of its constituents—for example, by incorporating the coupling of essential RNAs into a positive feedback loop. Without this feedback loop, the replicating system would be lost. These positive feedback loops formed the first hypercycles.

In the process described above, the fact that the first hypercycles originated from the quasispecies population (a population of similar sequences) created a significant advantage. One possibility of linking different chains *I*—which is relatively easy to achieve, taking into account the quasispecies properties—is that the one chain *I* improves the synthesis of the similar chain *I*’. In this way, the existence of similar sequences *I* originating from the same quasispecies population promotes the creation of the linkage between molecules *I* and *I*’.

### Evolutionary dynamics

After formation, a hypercycle reaches either an internal equilibrium or a state with oscillating concentrations of each type of chain I, but with the total concentration of all chains remaining constant. In this way, the system consisting of all chains can be expressed as a single, integrated entity. During the formation of hypercycles, several of them could be present in comparable concentrations, but very soon, a selection of the hypercycle with the highest fitness value will take place [1]. Here, the fitness value expresses the adaptation of the hypercycle to the environment, and the selection based on it is very sharp. After one hypercycle wins the competition, it is very unlikely that another one could take its place, even if the new hypercycle would be more efficient than the winner. Usually, even large fluctuations in the numbers of internal species cannot weaken the hypercycle enough to destroy it. In the case of a hypercycle, we can speak of one-for-ever selection, which is responsible for the existence of a unique translation code and a particular chirality [2].

The above-described idea of a hypercycle’s robustness results from an exponential growth of its constituents caused by the catalytic support. However, Eörs Szathmáry and Irina Gladkih showed that an unconditional coexistence can be obtained even in the case of a non-enzymatic template replication that leads to a subexponential or a parabolic growth [26]. This could be observed during the stages preceding a catalytic replication that are necessary for the formation of hypercycles. The coexistence of various non-enzymatically replicating sequences could help to maintain a sufficient diversity of RNA modules used later to build molecules with catalytic functions.

From the mathematical point of view, it is possible to find conditions required for cooperation of several hypercycles. However, in reality, the cooperation of hypercycles would be extremely difficult, because it requires the existence of a complicated multi-step biochemical mechanism or an incorporation of more than two types of molecules. Both conditions seem very improbable; therefore, the existence of coupled hypercycles is assumed impossible in practice.

Evolution of a hypercycle ensues from the creation of new components by the mutation of its internal species. Mutations can be incorporated into the hypercycle, enlarging it if, and only if, two requirements are satisfied. First, a new information carrier *I*_*new*_ created by the mutation must be better recognized by one of the hypercycle's members *I*_*i*_ than the chain *I*_i+1_ that was previously recognized by it. Secondly, the new member *I*_*new*_ of the cycle has to better catalyse the formation of the polynucleotide *I*_i+1_ that was previously catalysed by the product of its predecessor *I*_*i*_. In theory, it is possible to incorporate into the hypercycle mutations that do not satisfy the second condition. They would form parasitic branches that use the system for their own replication but do not contribute to the system as a whole. However, it was noticed that such mutants do not pose a threat to the hypercycle, because other constituents of the hypercycle grow nonlinearly, which prevents the parasitic branches from growing.

### Evolutionary dynamics: a mathematical model

According to the definition of a hypercycle, it is a nonlinear, dynamic system, and, in the simplest case, it can be assumed that it grows at a rate determined by a system of quadratic differential equations. Then, the competition between evolving hypercycles can be modelled using the differential equation

$${\dot{C}}_{l}=q_{l}C_{l}^{2}-C_{l}\frac{\phi }{C}$$

where

$$C=\sum_{l}C_{l}.$$

Here, *C*_*l*_ is the total concentration of all polynucleotide chains belonging to a hypercycle *H*_*l*_, C is the total concentration of polynucleotide chains belonging to all hypercycles, *q*_*l*_ is the rate of growth, and *ϕ* is a dilution flux that guarantees that the total concentration is constant. According to the above model, in the initial phase, when several hypercycles exist, the selection of the hypercycle with the largest *q*_*l*_ value takes place. When one hypercycle wins the selection and dominates the population, it is very difficult to replace it, even with a hypercycle with a much higher growth rate *q*.

## Compartmentalization and Genome Integration

Hypercycle theory proposed that hypercycles are not the final state of organization, and further development of more complicated systems is possible by enveloping the hypercycle in some kind of membrane [2,3]. After evolution of compartments, a genome integration of the hypercycle can proceed by linking its members into a single chain, which forms a precursor of a genome. After that, the whole individualized and compartmentalized hypercycle can behave like a simple self-replicating entity. Compartmentalization provides some advantages for a system that has already established a linkage between units. Without compartments, genome integration would boost competition by limiting space and resources. Moreover, adaptive evolution requires the package of transmissible information for advantageous mutations in order not to aid less-efficient copies of the gene. The first advantage is that it maintains a high local concentration of molecules, which helps to locally increase the rate of synthesis. Secondly, it keeps the effect of mutations local, while at the same time affecting the whole compartment. This favours preservation of beneficial mutations, because it prevents them from spreading away. At the same time, harmful mutations cannot pollute the entire system if they are enclosed by the membrane. Instead, only the contaminated compartment is destroyed, without affecting other compartments. In that way, compartmentalization allows for selection for genotypic mutations. Thirdly, membranes protect against environmental factors because they constitute a barrier for high-weight molecules or UV irradiation. Finally, the membrane surface can work as a catalyst.

Despite the above-mentioned advantages, there are also potential problems connected to compartmentalized hypercycles. These problems include difficulty in the transport of ingredients in and out, synchronizing the synthesis of new copies of the hypercycle constituents, and division of the growing compartment linked to a packing problem.

In the initial works, the compartmentalization was stated as an evolutionary consequence of the hypercyclic organization. Carsten Bresch and coworkers raised an objection that hypercyclic organization is not necessary if compartments are taken into account [27]. They proposed the so-called package model, in which one type of a polymerase is sufficient and copies all polynucleotide chains that contain a special recognition motif. However, as pointed out by the authors, such packages are—contrary to hypercycles—vulnerable to deleterious mutations as well as a fluctuation abyss, resulting in packages that lack one of the essential RNA molecules. Eigen and colleagues argued that simple package of genes cannot solve the information integration problem and hypercycles cannot be simply replaced by compartments, but compartments may assist hypercycles [28]. This problem, however, raised more objections, and Eörs Szathmáry and László Demeter reconsidered whether packing hypercycles into compartments is a necessary intermediate stage of the evolution. They invented a stochastic corrector model [10] that assumed that replicative templates compete within compartments, and selective values of these compartments depend on the internal composition of templates. Numerical simulations showed that when stochastic effects are taken into account, compartmentalization is sufficient to integrate information dispersed in competitive replicators without the need for hypercycle organization. Moreover, it was shown that compartmentalized hypercycles are more sensitive to the input of deleterious mutations than a simple package of competing genes [29]. Nevertheless, package models do not solve the error threshold problem that originally motivated the hypercycle.

## Hypercycles and Ribozymes

At the time of the hypercycle theory formulation, ribozymes were not known. After the breakthrough of discovering RNA’s catalytic properties in 1982 [24,25], it was realized that RNA had the ability to integrate protein and nucleotide-chain properties into one entity. Ribozymes potentially serving as templates and catalysers of replication can be considered components of quasispecies that can self-organize into a hypercycle without the need to invent a translation process. In 2001, a partial RNA polymerase ribozyme was designed via directed evolution [30]. Nevertheless, it was able to catalyse only a polymerization of a chain having the size of about 14 nucleotides, even though it was 200 nucleotides long. The most up-to-date version of this polymerase was shown in 2015 [31]. While it has an ability to catalyse polymerization of longer sequences, even of its own length, it cannot replicate itself. In 2014, a cross-chiral RNA polymerase ribozyme was demonstrated [32]. It was hypothesized that it offers a new mode of recognition between an enzyme and substrates, which is based on the shape of the substrate, and allows avoiding the Watson-Crick pairing and, therefore, may provide greater sequence generality. Various other experiments have shown that, besides bearing polymerase properties, ribozymes could have developed other kinds of evolutionarily useful catalytic activity, such as synthase, ligase, or aminoacylase activities [30]. Ribozymal aminoacylators and ribozymes with the ability to form peptide bonds might have been crucial to inventing translation. An RNA ligase, in turn, could link various components of quasispecies into one chain, beginning the process of a genome integration. An RNA with a synthase or a synthetase activity could be critical for building compartments and providing building blocks for growing RNA and protein chains as well as other types of molecules. Many examples of this kind of ribozyme are currently known, including a peptidyl transferase ribozyme [33], a ligase [34,35], and a nucleotide synthetase [36]. A transaminoacylator described in 2013 has five nucleotides [37], which is sufficient for a trans-amino acylation reaction and makes it the smallest ribozyme that has been discovered. It supports a peptidyl-RNA synthesis that could be a precursor for the contemporary process of linking amino acids to tRNA molecules. An RNA ligase’s catalytic domain, consisting of 93 nucleotides, proved to be sufficient to catalyse a linking reaction between two RNA chains [38]. Similarly, an acyltransferase ribozyme 82 nucleotides long was sufficient to perform an acyltransfer reaction [39]. Altogether, the results concerning the RNA ligase’s catalytic domain and the acyltransferase ribozyme are in agreement with the estimated upper limit of 100 nucleotides set by the error threshold problem. However, it was hypothesized that even if the putative first RNA-dependent RNA-polymerases are estimated to be longer—the smallest reported up-to-date RNA-dependent polymerase ribozyme is 165 nucleotides long [30]—they did not have to arise in one step [40]. It is more plausible that ligation of smaller RNA chains performed by the first RNA ligases resulted in a longer chain with the desired catalytically active polymerase domain.

Forty years after the publication of Manfred Eigen’s primary work dedicated to hypercycles, Nilesh Vaidya and colleagues showed experimentally that ribozymes can form catalytic cycles and networks capable of expanding their sizes by incorporating new members [14]. However, this is not a demonstration of a hypercycle in accordance with its definition, but an example of a collectively autocatalytic set [15]. Earlier computer simulations showed that molecular networks can arise, evolve, and be resistant to parasitic RNA branches [41]. In their experiments, Vaidya et al. used an Azoarcus group I intron ribozyme that, when fragmented, has an ability to self-assemble by catalysing recombination reactions in an autocatalytic manner. They mutated the three-nucleotide-long sequences responsible for recognition of target sequences on the opposite end of the ribozyme (namely, Internal Guide Sequences or IGSs) as well as these target sequences. Some genotypes could introduce cooperation by recognizing target sequences of the other ribozymes, promoting their covalent binding, while other selfish genotypes were only able to self-assemble. In separation, the selfish subsystem grew faster than the cooperative one. After mixing selfish ribozymes with cooperative ones, the emergence of cooperative behaviour in a merged population was observed, outperforming the self-assembling subsystems. Moreover, the selfish ribozymes were integrated into the network of reactions, supporting its growth. These results were also explained analytically by the ODE model and its analysis. They differ substantially from results obtained in evolutionary dynamics [42]. According to evolutionary dynamics theory, selfish molecules should dominate the system even if the growth rate of the selfish subsystem in isolation is lower than the growth rate of the cooperative system. Moreover, Vaidya et al. proved that, when fragmented into more pieces, ribozymes that are capable of self-assembly can not only still form catalytic cycles but, indeed, favour them. Results obtained from experiments by Vaidya et al. gave a glimpse on how inefficient prebiotic polymerases, capable of synthesizing only short oligomers, could be sufficient at the pre-life stage to spark off life. This could happen because coupling the synthesis of short RNA fragments by the first ribozymal polymerases to a system capable of self-assembly not only enables building longer sequences but also allows exploiting the fitness space more efficiently with the use of the recombination process. Another experiment performed by Hannes Mutschler et al. [31] showed that the RNA polymerase ribozyme, which they described, can be synthesized in situ from the ligation of four smaller fragments, akin to a recombination of Azoarcus ribozyme from four inactive oligonucleotide fragments described earlier [43]. Apart from a substantial contribution of the above experiments to the research on the origin of life, they have not proven the existence of hypercycles experimentally.

## Related Problems and Reformulations

The hypercycle concept has been continuously studied since its origin. Shortly after Eigen and Schuster published their main work regarding hypercycles, John Maynard Smith raised an objection that the catalytic support for the replication given to other molecules is altruistic [44]. Therefore, it cannot be selected and maintained in a system. He also underlined hypercycle vulnerability to parasites, as they are favoured by selection. Later on, Josef Hofbauer and Karl Sigmund [45] indicated that, in reality, a hypercycle can maintain only fewer than five members. In agreement with Eigen and Schuster’s principal analysis, they argued that systems with five or more species exhibit limited and unstable cyclic behaviour, because some species can die out due to stochastic events and break the positive feedback loop that sustains the hypercycle. The extinction of the hypercycle then follows. It was also emphasized that a hypercycle size of up to four is too small to maintain the amount of information sufficient to cross the information threshold.

Several researchers proposed a solution to these problems by introducing space into the initial model either explicitly [6,13,46,47] or in the form of a spatial segregation within compartments [10,27]. Bresch et al. [27] proposed a package model as a solution for the parasite problem. Later on, Szathmáry and Demeter [10] proposed a stochastic corrector machine model. Both compartmentalized systems proved to be robust against parasites. However, package models do not solve the error threshold problem that originally motivated the idea of the hypercycle. A few years later, Maarten Boerlijst and Paulien Hogeweg, and later Nobuto Takeuchi, studied the replicator equations with the use of partial differential equations [5] and cellular automata models [6,7,9], methods that already proved to be successful in other applications [48,49]. They demonstrated that spatial self-structuring of the system completely solves the problem of global extinction for large systems and, partially, the problem of parasites [13]. The latter was also analysed by Robert May, who noticed that an emergent rotating spiral wave pattern, which was observed during computational simulations performed on cellular automata, proved to be stable and able to survive the invasion of parasites if they appear at some distance from the wave core [12]. Unfortunately, in this case, rotation decelerates as the number of hypercycle members increases, meaning that selection tends toward decreasing the amount of information stored in the hypercycle. Moreover, there is also a problem with adding new information into the system. In order to be preserved, the new information has to appear near to the core of the spiral wave. However, this would make the system vulnerable to parasites, and, as a consequence, the hypercycle would not be stable. Therefore, stable spiral waves are characterized by once-for-ever selection, which creates the restrictions that, on the one hand, once the information is added to the system, it cannot be easily abandoned; and on the other hand, new information cannot be added.

Another model based on cellular automata, taking into account a simpler replicating network of continuously mutating parasites and their interactions with one replicase species, was proposed by Takeuchi and Hogeweg [7] and exhibited an emergent travelling wave pattern. Surprisingly, travelling waves not only proved to be stable against moderately strong parasites, if the parasites’ mutation rate is not too high, but the emergent pattern itself was generated as a result of interactions between parasites and replicase species. The same technique was used to model systems that include formation of complexes [50]. Finally, hypercycle simulation extending to three dimensions showed the emergence of the three-dimensional analogue of a spiral wave, namely, the scroll wave [51].

The version history of the text file and the peer reviews (and response to reviews) are available as supporting information in S1 and S2 Texts.

## Supporting Information

S1 Text Version history of the text file. (XML)

S2 Text Peer reviews and response to reviews. Human-readable versions of the reviews and authors' responses are available as comments on this article.
(XML)
